# Rituximab efficiently depletes B cells in lung tumors and normal lung tissue

**DOI:** 10.12688/f1000research.7599.1

**Published:** 2016-01-08

**Authors:** Albane Joly-Battaglini, Clara Hammarström, Branislava Stankovic, Henrik Aamodt, Johan Stjärne, Odd Terje Brustugun, Åslaug Helland, Inger Øynebråten, Alexandre Corthay

**Affiliations:** 1 Tumor Immunology group, Department of Pathology, Oslo University Hospital, Oslo, Norway; 2 Centre for Immune Regulation, University of Oslo, Oslo, Norway; 3 Department of Pathology, Oslo University Hospital, Oslo, Norway; 4 Department of Cardiothoracic Surgery, Oslo University Hospital, Oslo, Norway; 5 Betanien Hospital, Skien, Norway; 6 Department of Oncology, Norwegian Radium Hospital, Oslo University Hospital, Oslo, Norway; 7 Department of Cancer Genetics, Institute for Cancer Research, Norwegian Radium Hospital, Oslo University Hospital, Oslo, Norway; 8 Department of Biosciences, University of Oslo, Oslo, Norway

**Keywords:** rituximab, B cells, depletion, monoclonal antibody, lungs, tumor, lymph node, non-small cell lung cancer

## Abstract

Rituximab is a monoclonal antibody that targets the CD20 B-cell-specific antigen and is widely used as therapy for B-cell lymphoma. Since rituximab depletes both malignant and normal B cells, it is increasingly being used to treat various conditions in which normal B cells have a pathogenic role, such as rheumatoid arthritis and multiple sclerosis. It is well-established that rituximab efficiently eliminates B cells in blood, lymph nodes, and spleen. In contrast, the effect of rituximab in non-lymphoid tissues remains poorly documented and is debated. Here, we report a rheumatoid arthritis patient who was treated with rituximab before receiving thoracic surgery for non-small cell lung cancer. Using flow cytometry and immunohistochemistry, we show that rituximab efficiently depleted CD20-positive B cells in a primary lung tumor, in lung-associated lymph nodes, and in normal lung tissue. We conclude that rituximab may be very efficient at depleting normal B cells in the lungs. This property of rituximab may potentially be exploited for the treatment of conditions in which pathogenic B cells reside in the lungs. On the other hand, the clearance of lung B cells may provide an explanation for the rare cases of severe non-infectious pulmonary toxicity of rituximab.

## Introduction

Rituximab was the first monoclonal antibody to be approved for the treatment of cancer and it is estimated that >4 million people have been treated with rituximab worldwide ^1^. Rituximab is a depleting chimeric anti-CD20 monoclonal antibody routinely used for the treatment of B-cell lymphoma ^2– 4^. The B cell-specific antigen CD20 is expressed on all normal B cells, except for early B cell precursors and antibody-secreting plasma cells, and by nearly all B-cell lymphomas. Since rituximab depletes both malignant and normal B cells, its use has been extended to non-cancerous conditions in which normal B cells are believed to play a central role in pathogenesis. Significant clinical benefits have been reported for the treatment of autoimmune diseases, such as rheumatoid arthritis, multiple sclerosis, vasculitis, Sjögren’s syndrome, and scleroderma ^5– 9^. The mechanism whereby rituximab depletes B cells is not fully understood but there is evidence for complement-dependent cell lysis and for antibody-dependent cellular cytotoxicity ^2, 10, 11^. It has been shown that rituximab efficiently eliminates normal and malignant B cells in blood and in lymphoid organs such as lymph nodes, spleen, and bone marrow ^12– 14^. In contrast, the effect in non-lymphoid tissues remains poorly documented. Here, we report the effect of rituximab in the lungs of a patient who was treated with rituximab because of rheumatoid arthritis before receiving thoracic surgery for non-small cell lung cancer.

## Methods

### Ethics approval

The Regional Committee for Medical and Health Research Ethics (Oslo, Norway) has approved the study (permit number: REK S-05307). Written informed consent for publication of the clinical details was obtained from all patients included in the study.

### Flow cytometry

Patient blood was sampled from a central venous catheter before the start of surgery and collected into ethylenediaminetetraacetic acid (EDTA)-containing tubes. Peripheral blood mononuclear cells (PBMCs) were isolated using a gradient (Lymphoprep, Axis-Shield, Oslo, Norway; cat. no. 1114544). Fresh biopsies from the tumor, a lung-associated lymph node, and normal lung tissue, were sampled under sterile conditions in the operating room, after the removal of the lung lobe by the surgeon. Samples were treated enzymatically with 2 mg/ml collagenase A and 50 units/ml DNase (both from Roche, Basel, Switzerland; collagenase A, cat. no. 10103586001; DNase, cat. no. 11284932001) and incubated for 1 h on a magnet stirrer at 37°C. Single-cell suspension was obtained by squeezing the dissolved tissue through a 100 μm mesh and centrifuging at 300g for 7 min. Nonspecific binding was blocked by incubation with 12.5 μg/ml IgG purified from pooled mouse sera (Sigma-Aldrich, St. Louis, Missouri, USA; cat. no. I8765). Cells were stained in a 96-well plate for 20 min on ice with fluorochrome-labeled monoclonal antibodies diluted 1:10 in phosphate-buffered saline (Sigma-Aldrich, cat. no. D8537) supplemented with 10% foetal bovine serum (Sigma-Aldrich cat. no. F7524). The following monoclonal antibodies were used (all from BioLegend, San Diego, California, USA): anti-CD3 (clone UCHT1, cat. no. 300415); anti-CD4 (clone OKT4, cat. no. 317409); anti-CD8 (clone SK1, cat. no. 344713); anti-CD14 (clone HCD14, cat. no. 325617); anti-CD19 (clone HIB19, cat. no. 302227); anti-CD45 (clone HI30, cat. no. 304029); anti-HLA-DR (clone L243, cat. no. 307610). Stained cells were analyzed with a BD LSRFortessa ^TM^ Cell Analyzer instrument (BD Biosciences, Franklin Lakes, New Jersey, USA, model no. 647794E6) and FlowJo software version 10 (FlowJo, Ashland, Oregon, USA).

### Tissue preparation and immunohistochemistry

For light microscopy, 4 μm thick sections from formalin-fixed paraffin-embedded tissue were automatically stained with hematoxylin and eosin in a Sakura Tissue-Tek Prisma instrument (Sakura Finetek, Torrance, California, USA). The immunostainings were done on a Dako Autostainer instrument (Dako, Agilent Technologies, Santa Clara, California, USA, model Link 48), and the incubation time for the primary antibodies was 20 min. CD3 was immunostained by clone SP7 (a monoclonal rabbit antibody, diluted 1:150; Thermo Scientific, Waltham, Massachusetts, USA; cat. no. RM-9107), and CD20 was immunostained by clone L26 (a mouse IgG2a antibody, diluted 1:600; Dako, cat. no. M0755). The secondary detection was performed with Dako EnVision ^TM^ Flex (Dako, cat. no. K8000) for 20 min, followed by diaminobenzidine (DAB) staining for 10 min. The slides were thereafter treated with CuSO4 for 5 min before contrastaining with hematoxylin. Samples were examined with a Nikon Eclipse model N *i*-U microscope (Nikon, Tokyo, Japan) equipped with Nikon Plan-Fluor objective lenses (2×, 20×, and 40×) and images were taken with an Infinity 2 digital camera (Lumenera Corporation, Nepean, Ontario, Canada).

## Results

A 62-year-old woman with seronegative rheumatoid arthritis was diagnosed in 2015 with lung adenocarcinoma, stage IIB (pT3N0Mx, TNM 7 ^th^ edition). The patient, a former heavy-smoker with a smoking history of 30 pack-years, underwent right lower lobectomy. The patient had been treated with Prednisolone (usually 5 mg daily since 2005), as well as several different drugs (Methotrexate 10 mg/week for 3 weeks in 2005, Metoject 1×10 mg in 2005 and 2×10mg in 2007, Plaquenil 400 mg/day in January-February 2006, Arava 10 mg/day for 8 days in 2006, and Enbrel 50 mg/week from January 2008 to April 2009), all discontinued due to side-effects or inefficiency. Over the past six years before lung cancer diagnosis (2009–2014), the patient received seven cycles of rituximab (MabThera, 6 cycles of 2×1000mg and 1 cycle of 2×500mg) with only moderate clinical effect. Serum immunoglobulin (Ig) levels were normal before initiation of the rituximab treatment (IgG=6.1g/L; IgA=1.3g/L; IgM=2.1g/L), excluding any B-cell immunodeficiency. Serum IgA and IgM levels remained normal (IgA≥0.9g/L; IgM≥1.2g/L), whereas low IgG levels (4.2–5.6g/L) were observed several times over the past three years. The last rituximab cycle (2×1000mg) was given 6 months pre-operatively.

**Figure 1.**
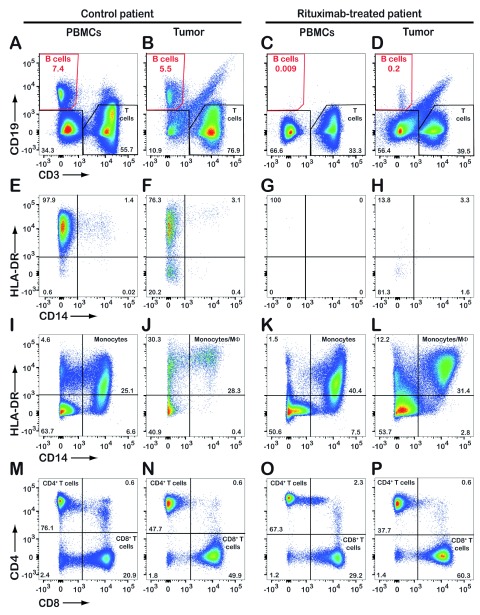
Flow cytometric analysis reveals the absence of tumor-infiltrating CD19 ^+^ B cells in a patient treated with rituximab. Upon chest surgery for removal of a lung tumor from a patient previously treated with rituximab, tumor biopsy and serum samples were analyzed by flow cytometry. Results from a control lung cancer patient (not treated with rituximab) are shown for comparison. Both patients were diagnosed with lung adenocarcinoma. CD45-positive leukocytes were gated and analyzed further for expression of CD19/CD3 ( **A**– **D**), HLA-DR/CD14 ( **E**– **L**), and CD4/CD8 ( **M**– **P**). The dot plots **E**– **H** show expression of HLA-DR and CD14 by CD19-positive B cells only (red gates in **A**– **D**). Numbers in quadrants indicate the percentage of cells detected. PBMCs, peripheral blood mononuclear cells.

**Figure 2.**
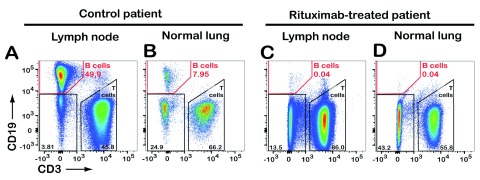
Rituximab depletes B cells in lung-associated lymph node and normal lung tissue. Upon chest surgery for removal of tumor-containing lung lobe from a patient previously treated with rituximab, a lung-associated lymph node and normal lung tissue samples were analyzed by flow cytometry. Results from a control lung cancer patient (not treated with rituximab) are shown for comparison. Both patients were diagnosed with lung adenocarcinoma. Live leukocytes (CD45-positive, propidium iodide-negative) were gated and analyzed further for expression of CD19 (B cells) and CD3 (T cells). Numbers indicate the percentage of cells detected in each gate.

Upon informed consent, the patient was included in a research project. A pre-operative blood sample and biopsy samples from the tumor, a lung-associated lymph node, and normal lung were collected for flow cytometric analysis. For comparison, samples from a control patient with lung adenocarcinoma (not treated with rituximab) were analyzed. The control patient had CD19 ^+^ B cells in blood and tumor ( Figure 1A,B). In the rituximab-treated patient, CD19 ^+^ B cells were virtually absent from the blood ( Figure 1C) and strongly reduced in the tumor (0.2% of all CD45-positive leukocytes, Figure 1D). The remaining CD19 ^+^ B cells in the tumor were mostly HLA-DR-negative ( Figure 1H) indicating that they were plasma cells which typically lack the CD20 antigen. Rituximab did not deplete other types of immune cells, such as monocytes/macrophages, CD4 ^+^ T cells, or CD8 ^+^ T cells ( Figure 1I–P). Flow cytometric analysis of a lung-associated lymph node and normal lung tissue revealed virtual absence of CD19 ^+^ B cells in the rituximab-treated patient ( Figure 2).

**Figure 3.**
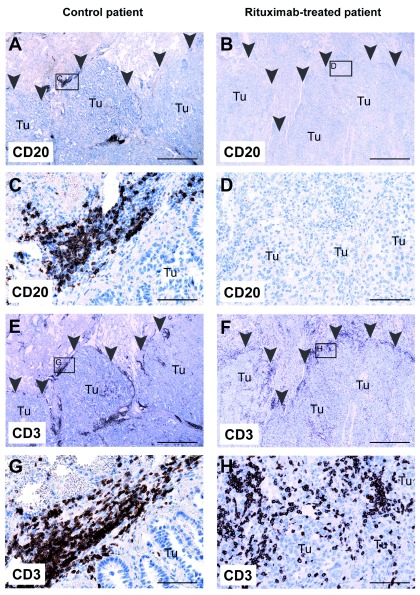
Absence of CD20 ^+^ B cells in primary lung tumor from a patient treated with rituximab. Lung tissue sections from a control patient ( *left*) and from a rituximab-treated patient ( *right*) were stained with anti-CD20 ( **A**– **D**) or anti-CD3 ( **E**– **H**) antibodies, and contrastained with hematoxylin. Both patients were diagnosed with lung adenocarcinoma. Arrowheads delineate the border of the tumor. Small boxes in **A**, **B**, **E**, and **F** indicate magnified areas in **C**, **D**, **G**, and **H**, respectively. Tu, tumor tissue. **A**, **B**, **E**, and **F**: 20× magnification; scalebar = 1 mm. **C**, **D**, **G**, and **H**: 200× magnification; scalebar = 100 μm.

Immunohistochemistry of formalin-fixed paraffin-embedded routine specimen was performed by staining for CD3 and CD20. In the control patient, the inflammatory infiltrate in and around the tumor contained both CD20 ^+^ B cells ( Figure 3A, C) and CD3 ^+^ T cells ( Figure 3E, G). In contrast, the inflammatory infiltrate in the rituximab-treated patient contained T cells ( Figure 3F, H) but virtually no B cells ( Figure 3B, D), in accordance with the flow cytometry data.

**Figure 4.**
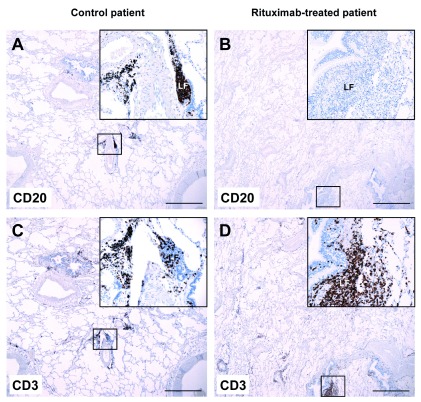
Absence of CD20 ^+^ B cells in normal lung tissue from a patient treated with rituximab. Normal lung tissue sections from a control patient ( *left*) and from a rituximab-treated patient ( *right*) were stained with anti-CD20 ( **A**, **B**) or anti-CD3 ( **C**, **D**) antibodies, and contrastained with hematoxylin. Both patients were diagnosed with lung adenocarcinoma. Small boxes indicate areas that are magnified in the upper right inserts. LF, peribronchial lymphoid focus. Main images: 20× magnification; scalebar = 1 mm. Upper right inserts: 200× magnification.

**Figure 5.**
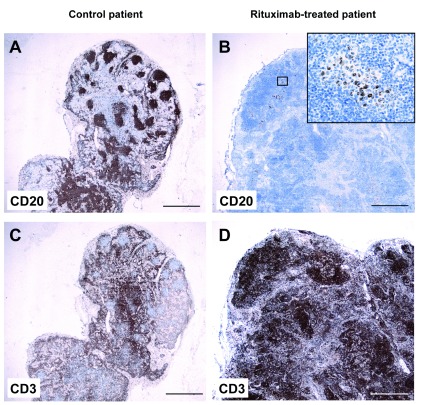
Absence of CD20 ^+^ B cells in lung-associated lymph node from a patient treated with rituximab. A lung-associated lymph node from a control patient ( *left*) and from a rituximab-treated patient ( *right*) were stained with anti-CD20 ( **A**– **B**) or anti-CD3 ( **C**– **D**) antibodies, and contrastained with hematoxylin. Both patients were diagnosed with lung adenocarcinoma. The small box in **B** indicates the area that is magnified in the upper right insert. The black dots in **B** represent anthracotic pigment in macrophages. Main images: 20× magnification; scalebar = 1 mm. Upper right insert in **B**: 400× magnification.

The same pattern was observed in normal lung and in lung-associated lymph nodes. In the control patient, normal lung tissue contained peribronchial lymphoid foci with both CD20 ^+^ B cells ( Figure 4A) and CD3 ^+^ T cells ( Figure 4C). In contrast, the peribronchial lymphoid foci from the rituximab-treated patient contained T cells ( Figure 4D) but virtually no B cells ( Figure 4B). In control lung-associated lymph nodes, a normal lymphocyte distribution was observed with typical germinal centers with a high density of CD20 ^+^ B cells ( Figure 5A), whereas CD3 ^+^ T cells were mostly present outside the germinal centers ( Figure 5C). In sharp contrast, lung-associated lymph nodes from the rituximab-treated patient contained virtually no B cells ( Figure 5B) and were homogenously and densely populated by T cells ( Figure 5D). The black dots in Figure 5B represent anthracotic pigment in macrophages. Thus, rituximab therapy resulted in efficient depletion of CD20-positive B cells throughout the lungs, including in a lung tumor, in normal lung tissue, and in lung-associated lymph nodes.

## Discussion

Rituximab was initially developed with the goal of eradicating B-lymphoma cells which typically reside in blood and lymphoid organs ^1– 4^. It is now well established that rituximab efficiently eliminates normal and malignant B cells in those anatomical locations ^12– 14^. In contrast, current knowledge on the effect of rituximab therapy in non-lymphoid tissues remains fragmentary. This is problematic because rituximab is being considered as a therapeutic option for a number of non-malignant conditions such as autoimmune diseases ^5– 9^ and myalgic encephalopathy/chronic fatigue syndrome ^15^. In autoimmune diseases, depletion of pathogenic B cells in inflamed tissues is likely to be required to obtain clinical benefits. In the cerebrospinal fluid of patients with multiple sclerosis, rituximab therapy was shown to result in 90–95% depletion of B cells ^16, 17^. In the salivary glands of patients with Sjögren’s syndrome, the efficiency of rituximab remains controversial because both complete and partial depletion of B cells have been reported ^8, 18^. Similarly, the effect of rituximab on synovial B cells is debated since various levels of depletion have been reported in patients with rheumatoid arthritis ^12, 19, 20^.

Our case report illustrates that rituximab may efficiently deplete B cells in the lungs, including lung tumor, normal lung tissue, and lung-associated lymph nodes. This property of rituximab is of particular interest for the treatment of conditions in which pathogenic B cells reside in the lungs, such as antisynthetase syndrome, granulomatosis with polyangiitis, and scleroderma-associated interstitial lung disease ^9, 21, 22^. On the other hand, the strong B cell-depleting effect in the lungs may provide an explanation for the rare cases of severe non-infectious pulmonary toxicity of rituximab ^23, 24^. Rituximab-associated lung disease is a rare but potentially fatal complication of rituximab therapy, whose pathogenic mechanism remains to be elucidated ^23^.

Rituximab therapy was associated with virtual absence of tumor-infiltrating B cells in a patient with lung adenocarcinoma. Non-small cell lung cancer (NSCLC) tumors typically contain tertiary lymphoid structures with a high frequency of CD20 ^+^ follicular B cells ^25^. Tumor-infiltrating immune cells, including B cells, may represent an ongoing protective immune response against the malignant cells ^26, 27^. In fact, it has recently been reported that a high density of follicular B cells correlated with longer patient survival in NSCLC ^25^. Therefore, rituximab-mediated depletion of tumor-infiltrating normal B cells may potentially have a detrimental impact on the antitumor immune response, particularly in NSCLC.
