# Supplementary material for: Rituximab efficiently depletes B cells in lung tumors and normal lung tissue
Source: F1000Res. 2016 Jan 8;5:38. [Version 1] doi: 10.12688/f1000research.7599.1 (PMC4813631; doi:10.12688/f1000research.7599.1)
Supplement: Peer review history

# Peer review: Referee response for version 1

**DOI:** 10.5256/f1000research.8182.r12922

**Published:** 2016-03-29

**Authors:** Hilde Schjerven, Etapong Fonabei Ayongaba

**Affiliations:** 1 Department of Laboratory Medicine, School of Medicine, University of California, San Francisco, San Francisco, CA, USA; 2 University of Oslo, Oslo, Norway

This well-written paper by Joly-Battaglini *et al. *describes a case study on the effect of Rituximab on B cells in the lung tissue and tumor microenvironment of a rheumatoid arthritis patient with lung adenocarcinoma. The authors employ the complementing techniques of flow cytometry and immunohistochemistry to show that Rituximab efficiently eliminates CD19+ B cells in the lungs and a majority of CD19+ B cells in the tumor microenvironment, without depleting other lymphoid cells which are not CD20+. Only a low percentage (0.2 %) of CD19+ cells were found in the tumor, and these were suggested to be plasma cells due to lack of HLA-DR by flow cytometry.

This paper confirms the efficiency of Rituximab, and adds new knowledge on its ability to eliminate B cells in lung tissue and tumor microenvironment. The questions asked by the study were approached using appropriate methods, and the paper is clearly written.

Suggestions for minor revisions;

 

1. It would help the clarity of the paper if the title would indicate that it was a case study. Also, in the title, lung tumor should be corrected to singular, not plural (current title: “lung tumors”)
2. It would help the reader if the authors would comment on the life span of B cells with respect to the duration of Rituximab treatment. It would be interesting to see a discussion on whether the low percentage of presumed plasma cells observed in the tumor were from post- or pre- Rituximab treatment. Does Rituximab reach and actively deplete intra-tissue B cells, or is the depletion a result of decreased levels of circulating B cells able to infiltrate tissues, and reflects a decrease due to the life-span (or resident time) of tissue-resident B cells?
3. What could the significance of the 0.2% of plasma cells be with respect to B cell implicated diseases? This could be discussed in light of serum IgG levels and statement of the patient displaying “only moderate clinical effect”. It is interesting in regards to the discussion of use of Rituximab for treatment of B-cell mediated diseases (non-cancer) and tissue-resident B cells as opposed to circulating of lymphoid tissue B cells (see point above).
4. What is the statement “The black dots in Figure 5B represent anthracotic pigment in macrophages” based on? Although plausible, a suitable control could for instance be isotype control stain to display this as background, or CD19 (vs e.g. CD11b or similar) stain using a different secondary detection method (that would not detect anthracotic pigment). Although such added experiments might be feasible (if more material is left), it is not required, as it will not affect the main message of the paper in regards to the observed major depletion of B cells.

Overall, the article is well-written, scientifically sound, and presents a case study observation that warrants indexing.

We have read this submission. We believe that we have an appropriate level of expertise to confirm that it is of an acceptable scientific standard.

# Peer review: Referee response for version 1

**DOI:** 10.5256/f1000research.8182.r12242

**Published:** 2016-02-03

**Authors:** Bertrand Huard

**Affiliations:** 1 Department of Oncogenesis and Biotechnology, Joseph Fourier University, Grenoble, France

Title/abstract, content, conclusions, data are all appropriate.

As a minor revision, I would ask authors to speculate on what could be the function of B cells in non infectious lung.

I have read this submission. I believe that I have an appropriate level of expertise to confirm that it is of an acceptable scientific standard.
